# “It is not a scientific number it is just a feeling”: Populating a multi‐dimensional end‐of‐life decision framework using deliberative methods

**DOI:** 10.1002/hec.4239

**Published:** 2021-03-01

**Authors:** Joanna Coast, Cara Bailey, Alastair Canaway, Philip Kinghorn

**Affiliations:** ^1^ Health Economics Bristol Population Health Sciences Bristol Medical School University of Bristol Bristol UK; ^2^ School of Nursing Institute of Clinical Sciences University of Birmingham Birmingham UK; ^3^ Warwick Clinical Trials Warwick Medical School University of Warwick Warwick UK; ^4^ Health Economics Unit Institute of Applied Health Research University of Birmingham Birmingham UK

**Keywords:** capabilities, deaths, externalities, health, normative criteria

## Abstract

The capability approach is potentially valuable for economic evaluation at the end of life because of its conceptualization of wellbeing as freedom and the potential for capturing outcomes for those at end of life and those close to persons at the end of life. For decision making, however, this information needs to be integrated into current evaluation paradigms. This research explored weights for an integrated economic evaluation framework using a deliberative approach. Twelve focus groups were held (38 members of the public, 29 “policy makers,” seven hospice volunteers); budget pie tasks were completed to generate weights. Constant comparison was used to analyze qualitative data, exploring principles behind individuals' weightings. Average weights elicited from members of the general population and policy makers for the importance that should be given to close persons (vs. patients) were very similar, at around 30%. A “sliding scale” of weights between health gain and the capability for a good death resulted from the policy maker and volunteer groups, with increasing weight given to the capability for a good death as the trajectory got closer to death. These weights can be used in developing a more comprehensive framework for economic evaluation at end of life.

## 1 INTRODUCTION

Evaluating the cost‐effectiveness of interventions at the end of life poses problems for health economists. The standard approach to economic evaluation measures the outcome of interventions only in terms of the gains in health that arise from that intervention (National Institute for Health and Care Excellence, 2013). The focus of end of life care, however, is the provision of palliative or supportive care. This is aimed at both patients and families, and while it does incorporate issues such as pain and symptom management, it also focuses on various aspects of support including psychological, social and spiritual (Department of Health, 2008; National Palliative and End of Life Care Partnership, 2015; NHS England, 2014). Currently, therefore, there is a conflict between the purposes that are advocated for end of life care, and the approach to assessing outcomes for cost‐effectiveness analysis. This conflict also exists among the views of experts, some of whom argue for the standard approach of focusing on health gain, but many of whom suggest it should not be the sole focus of analysis (Kinghorn & Coast, 2019). The implication of this conflict is that the interventions that are actually most efficient may not be those that are funded.

Given these concerns, alternative frameworks have been proposed for evaluating the cost‐effectiveness of interventions at the end of life (Coast, 2014; Coast, Bailey, Canaway, & Kinghorn, 2016). Work that has advocated going beyond health gain has been largely focused within an alternative extra‐welfarist/non‐welfarist normative approach that focuses on capability (Sen, 1993), including both the capabilities of those at the end of life and those close to the person at the end of life, and, for the person at the end of life specifically, the capability to experience a good death. The capability approach is potentially particularly valuable at the end of life because of its conceptualization of wellbeing as freedom and its respect for individual autonomy and human heterogeneity (Coast et al., 2016), factors which may be particularly important at the end of life. In a capability approach, the idea of wellbeing as freedoms that a person may or may not choose to take up, is of fundamental importance. In the end of life setting, for example, this may mean the provision of opportunities for involvement in decision making, or the ability to spend time with friends and family, even if the person at the end of life does not choose to take up these freedoms.

Such an integrated capability‐based framework is inevitably more complex than the standard “health maximization” approach to economic evaluation. It does, however, have the potential to provide greater conceptual and ethical legitimacy than the current approach because it includes other important outcomes at the end of life, as well as consideration of outcomes beyond the patient (Coast et al., 2008). While the importance of informal care for economic evaluation has been noted (Brouwer, 2006; Brouwer, 2019) in end of life care there is particular concern about impacts on family and friends (Canaway, Al‐Janabi, Kinghorn, Bailey, & Coast, 2017; Dumont, Dumont, & Mongeau, 2008); furthermore, it remains unclear as to how nonhealth benefits to those who care for, or about, a person should be incorporated into standard economic evaluation (Al‐Janabi, Flynn, & Coast, 2011).

### 1.1 An integrated capability framework for economic evaluation of end of life interventions

The integrated capability framework used in this research starts from a perspective that the aim of end of life care is to enable the achievement of a good death (Coast, 2014; Coast et al., 2016). A framework concentrating wholly on capability would set this against the opportunity to achieve a good life in the period prior to end of life. An alternative that is perhaps more relevant within current decision‐making paradigms, such as that advocated by the National Institute for Health and Care Excellence in the UK (National Institute for Health and Care Excellence, 2013), would be to see the focus of evaluation for earlier periods in a person's life in terms of health gain, and only shift toward a focus on the capability for a good death during the period at the end of life. It is clear, however, that there is not an instant shift from a focus on health gain to a good death and the framework reflects research from a number of sources (Gott, Ingleton, Bennett, & Gardiner, 2011; Lunney, Lynn, Foley, Lipson, & Guralnik, 2003; Lunney, Lynn, & Hogan, 2002; Murray, Kendall, Boyd, & Sheikh, 2005; Porock, Parker‐Oliver, Zweig, Rantz, & Petroski, 2003) in proposing a gradual shift between objectives, reflecting the often concurrent delivery of curative and palliative interventions (Coventry, Grande, Richards, & Todd, 2005; Lloyd, 2004; Shah et al., 2006). The emphasis on achieving a good death within this framework is also broadly conceived; it draws on the notion that the opportunity for a good death must be related both to the person at the end of life and those who are close to them (Field & Cassel, 1997; Kehl, 2006; Stroebe, Schut, & Stroebe, 2007).

**FIGURE 1:**
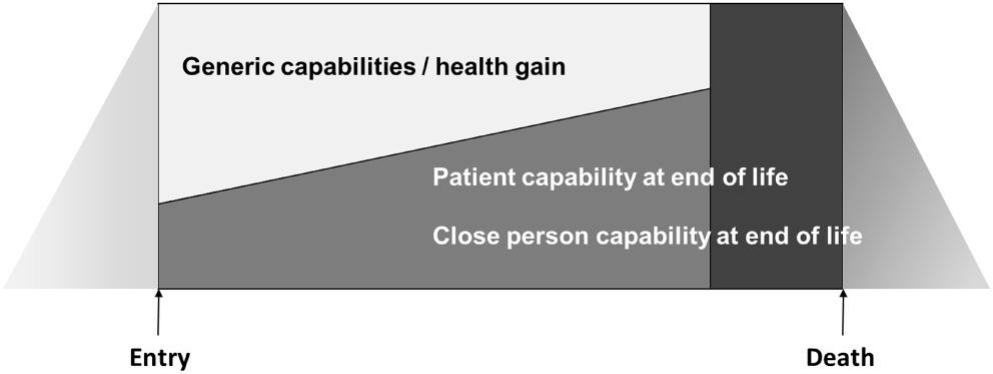
An integrated framework for decision making at the end of life as a person moves toward death: the period prior to entry represents increasing functional decline; “Entry” refers to the point at which end of life care becomes appropriate for the person, varying in time between individuals and causes of death; the light area represents the focus on health gain (or generic capabilities), the dark area the focus on a good death. The darkest area reflects the period of active dying, and the dark but fading (left to right) triangle, a period of bereavement of reducing intensity

Figure 1 shows the framework that will be explored further in this paper. Of particular interest for this paper are the two elements that focus on relative weights. The first is the relative importance to be placed on capabilities for the person at the end of life versus the importance to be placed on capabilities for close persons. The second is the relative importance to be placed on achieving health gain versus achieving the opportunity for a good death at various points along the trajectory toward death.

Obtaining explicit weights for these factors is needed if the framework is to be made workable. Such weights could come either through aggregation of individual weights or through some form of consensus approach (Coast et al., 2016). As developing such weights has not been attempted previously, the tasks were expected to be perceived as complex, and the work is necessarily exploratory. A deliberative approach (Burchardt, 2014; Fishkin & Luskin, 2005) provides a means of generating these values while providing the opportunity for discussion and debate both about the parameters generated and the nature of the task. A deliberative approach also fits well with Sen's views about the importance of public reasoning, debate and scrutiny in making social value judgments (Robeyns, 2005; Sen, 1999, 2005). Deliberation was here focused on external collective deliberation, taking place within small groups (Burkhalter, Gastil, & Kelshaw, 2002) and with the aim of generating free, open and reflective debate (Abelson, Blacksher, Li, Boesued, & Goold, 2013; Kinghorn, Canaway, Bailey, & Coast, 2017). This paper outlines the conduct of such a deliberative exercise and the findings obtained in relation to these weights for the integrated capability framework for end of life care.

## 2 METHODS

### 2.1 Study design

Two sets of focus group were undertaken with two groups of participants: members of the general population; and policy makers and service managers working in the area of end of life care. Ethics approval for the research was obtained from the University of Birmingham's Science, Technology, Engineering and Mathematics Ethical Review Committee (ERN_11‐1296).

### 2.2 Sampling

Members of the general population were sampled through purposeful random sampling (Owen‐Smith & Coast, 2017; Patton, 2015) using the edited electoral register and sampling across six electoral wards based on levels of deprivation, population density, palliative care provision and geographical considerations within one region of the UK. The only exclusion criterion was for those who had been bereaved within the last six months, for ethical reasons. Policy makers were recruited (i) through existing strategy groups, with the focus groups linked to existing meetings to facilitate getting participants together, and (ii) individually because of their particular role; individual policy makers all joined a single, separate group meeting. Groups of policy makers in the local area were identified through local nursing and commissioning group contacts; individual policy makers were identified through members of the research team, the broader project advisory group and relevant literature.

### 2.3 Data collection

Meetings for members of the general public were held within the relevant electoral ward in accessible local venues, with most groups taking place in the evening. Meetings with existing policy making groups were held prior to or following an adjoining meeting of the group, to maximize attendance. The meeting with individual policy makers was held in a central city location, convenient for national public transport. All group meetings were audio‐recorded and facilitated by at least three research staff including a lead facilitator, a co‐facilitator and note taker. All groups focused on decision‐making around end of life, but slightly different tasks were undertaken by each group type. Both members of the public and policy makers undertook initial tasks that familiarized them with two important aspects of the research tasks they would undertake in relation to generating weights for the integrated framework. These included tasks to familiarize participants with the separate capability end of life measures for individuals at the end of life and for close persons, and tasks to familiarize them with the methods used within the research.

There were two measures with which participants needed to be familiarized. The first was the ICECAP Supportive Care Measure (ICECAP‐SCM) (Huynh, Coast, Rose, Kinghorn, & Flynn, 2017; Sutton & Coast, 2014), a capability measure for individuals at the end of life comprising seven dimensions (choice, love and friendship, freedom from emotional suffering, freedom from physical suffering, dignity, support and preparation). The second was the ICECAP Close Person Measure (ICECAP‐CPM) (Canaway et al., 2017), a capability measure for close persons of individuals at the end of life comprising six dimensions (communication, practical support, privacy and space, emotional support, preparing and coping, emotional distress).

The method with which participants needed to be familiarized was a method used to obtain weights, referred to as the budget pie approach (Mullen, 1999). Using this approach, participants were presented with stylized pie charts and asked to allocate 100 tokens between the items included on the pie chart, as a means of expressing their weights.

Following the familiarization tasks, both group types (members of the general population and policy makers) were asked to complete a task to enable generation of weights between individuals at the end of life and close persons (task A). Policy maker groups only were also asked to complete a task focusing on generation of weights between health gain and a good death (task B).

#### 2.3.1 Task A: patients versus close persons

**FIGURE 2:**
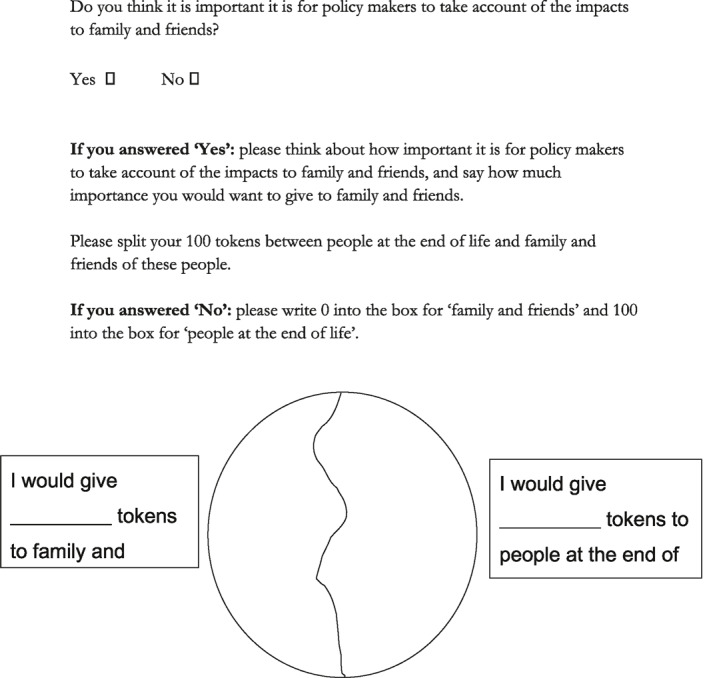
Task A—Taking account of impacts to family and friends

Using a budget pie approach, participants were presented with a pie chart with two segments. Participants were first asked “Do you think it is important for policy makers to take account of the benefits to family and friends?”. If they answered “yes,” participants were then asked to allocate their 100 tokens between “people at the end of life” and “family and friends” according to how much importance they would want to give to family and friends. In further clarification, participants were asked to consider the value of services and interventions, rather than their cost. Examples of the way in which the task was introduced are given in Boxes 1 and 2, and the task itself is shown in Figure 2.

Participants were asked initially to complete the task independently. Responses were then shared within the group and discussed. Discussion began by inviting participants to reveal their responses and to explore their reasoning, if they were happy to do so; discussion was then allowed to flow freely until it naturally ended. Participants were then given the opportunity to change their response following discussion if they so wished.

**BOX 1 Example explanation given for Task A to the general public**

The task was introduced verbally to each group. An example of the way in which this was done is given in full here:

Facilitator: This task is really shifting the focus to the decisions that policy makers have to go through in order to account for the impact on close persons of people who are dying. Now when I say impact, I really mean the, it's a range of experiences but things that could be simple like having somewhere nice to spend some quality time together with the person who's dying. It may be having the opportunity to sit down with a healthcare professional who's going to tell you about what's happening or preparing you for that, similar to what you said about when you were first told about the condition of your friend in D, actually that sense of time and was that an appropriate time and place and was that communication, the impact that had on you. It could be something a lot more complex about bereavement services that are available and the impact that those have or it may be about respite care for the person who's dying so that can have more of a positive impact on the person who was at home caring for them so all these different types of impacts. And on page 22 there's a question at the top there that actually says do you think it's important for policy makers to take account of these benefits, this impact on family and friends, do you think it's important. If you do think it's important that policy makers take into consideration family and friends as well as the patient, can you please tick yes. If you don't and you think it's more important that they focus entirely on the patient, please tick no. Now if you answered yes to that there's another circle at the bottom there actually asking you how much of your 100 tokens you would allocate to the family and friends and how much you would allocate to the person who's at the end of their life so if I could ask you if you have ticked yes there to allocate tokens, how many to the family and friends and how many tokens to the people at the end of life and write that figure in those boxes, again it should add up to 100. Now if you've ticked no at the top of that, that it’s not important for policy makers to take account of the benefits to family and friends of course you would give all of your 100 tokens to the person who's dying, the person at end of life and zero to the family and friends. Does that make sense?.

**BOX 2 Example explanation given for Task A to policy makers**

The task was introduced verbally to each group. An example of the way in which this was done is given in full here:

So now what we're going to do is think about moving a sort of level upwards. So, we've thought about how you might weight the different benefits for patients and how you might weight the different impacts or carers or family members, family carers. Now the question is, getting perhaps a little bit more difficult, how important is it, to include benefits to family and friends in your decision‐making? So, what we want to do now is think about whether you should take account of those benefits and if you are going to take account of those benefits, how much weight you would give to family and friends and how much weight you would give to patients. So, you're asked first of all to just decide whether or not you think policymakers should take account of impact to family and friends, so that's a simple yes/no. If you answer yes then, again, it's the hundred tokens, how many tokens would you give to family and friends? How many tokens would you give to people at the end of life? And if you say no, then can you just put a zero into the box for family and friends and a hundred into the box for people at the end of life.

#### 2.3.2 Task B: health gain versus a good death

**FIGURE 3:**
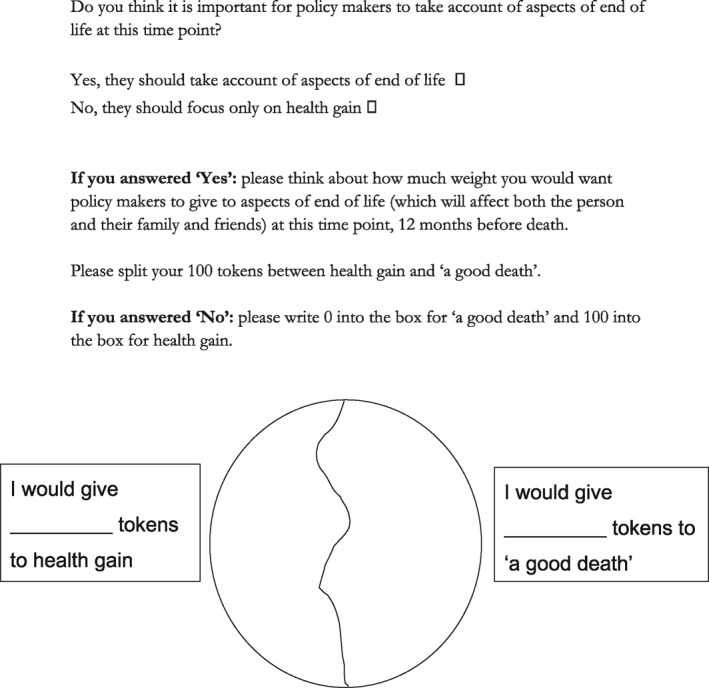
Task B—Taking account of “a good death” at 12 months before death

**FIGURE 4:**
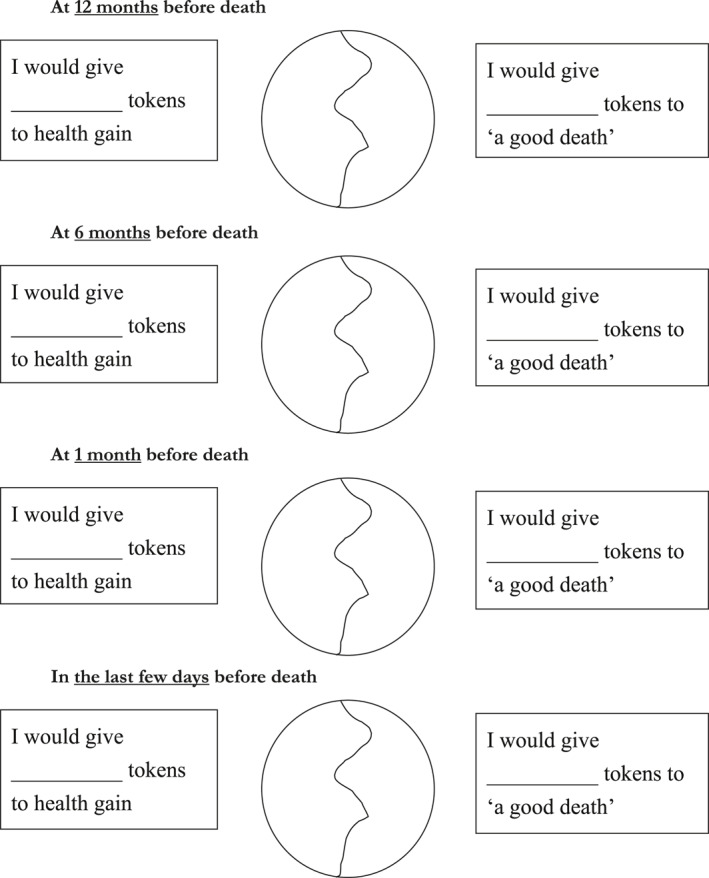
Task B —all included trajectories

Again using a budget pie approach, participants were presented with a series of pie charts, each with two segments and each representing a different time point before death (12 months before death, six months before death, one month before death, a few days before death); 12 months before death is sometimes used as a basis for thinking about end of life care (Marie Curie, 2018). Participants were first asked “Do you think it is important for policy makers to take account of aspects of end of life at this time point?”. If they answered “yes,” participants were then asked to allocate their 100 tokens between health gain and “a good death,” thinking about how much weight they would want policy makers to give to aspects of end of life (which will affect both the person and their family and friends) at that particular time point. An example of the way in which the task was asked is given in Box 3. The task for the 12‐month time period is shown in full in Figure 3, and the pie chart aspect of the task is shown for each time point in Figure 4. In presenting this task to participants, health gain was discussed in terms of the EQ‐5D (Brooks & EuroQol Group, 1996; Herdman et al., 2011), with its five dimensions of mobility, self‐care, usual activities, pain/discomfort and anxiety/depression, given that the work was conducted in the UK and EQ‐5D is the main measure recommended for use in decision making by the National Institute for Health and Care Excellence (NICE) (National Institute for Health and Care Excellence, 2018).

**BOX 3 Example explanation given for Task B (to policy makers)**

The task was introduced verbally to each group. An example of the way in which this was done is given in full here:

Facilitator: We're going to move on now to think about this question of, how important it is for policy makers to take account of people's deaths. We talked at the very beginning about whether end of life care was important and at the moment, decisions in NICE for example are made entirely on health gain, so interventions are only funded if they're cost effective in terms of providing health gain. What we've talked about in terms of these other measures is something a little bit broader than that, more around some sort of sense of a good death from the perspective of patients and their family and so that would be one option for thinking about outcomes, but if we're going to go down that route, then we have to think about how we would shift from a health gain measure to a measure of end of life and that's really what this next bit it asking you to do. So the sorts of measures that are used to measure health gain by NICE, look at things like mobility, self‐care, usual activities, pain and anxiety and they tend to be combined in a measure called QALY which some of you may be more familiar with than others. The alternative might be to shift toward these measures of a good death, of course death is very unpredictable for most people and we're aware that all deaths are very different so there is definitely a sense of artificiality in this next exercise that I'm going to ask you to do, but what we want you to do is sort of imagine, say the surprise question, you know, would you be surprised if this person was likely to die; this person died in the next 12 months, and what I want you to do is think about whether policy makers should take account of these end of life concerns or continue to focus purely on health gain, and again I'm going to ask you to split 100 tokens. So I'm going to ask you to do four exercises, one after the other, which ask you about different periods prior to death, so the first one is taking account of a good death at 12 months before death, and the question is, do you think it is important for policy makers to take account of aspects of end of life at this time point, yes or no. If you answer yes, then how much weight would you give to a good death and how much would you give to health gain, And if you answer no, then please write zero into the good death. And it's the same question but at different time points, so can I ask you to have a go at doing that exercise …

As with task A, participants initially completed the task independently and then had the opportunity to alter their response following discussion.

### 2.4 Data analysis

Responses from the workbooks were entered into a Microsoft Excel spreadsheet. Mean values and ranges were explored within each group, for the entire sample, and pre‐ and post‐discussion within the group.

Audio‐recordings from the focus groups were transcribed verbatim. Constant comparative analytical methods were used to provide an in‐depth assessment of discussion of the tasks (Glaser & Strauss, 1968; Strauss & Corbin, 1990). Open coding of the data relating to the completion of the two tasks provided a starting point for the analysis. An initial coding structure was developed and formed the basis for completion of separate analytic accounts related to each of the tasks and each group completing task A (Coast & Jackson, 2017). Quotes are used to illustrate the findings; quotes are presented verbatim with the use of ellipses to represent missing text; phrases such as “you know,” “like,” or “I mean,” as well as repeats of words that do not add to meaning, are excluded without use of ellipses. Information about the tokens allocated by participants from whom quotes are used is given following each quote, unless it is obvious within the quote.

## 3 RESULTS

### 3.1 Characteristics of the sample

Group meetings were held between June 2014 and March 2015.

#### 3.1.1 General population

A total of 2050 invitations were sent out across the six wards. These initial invitations resulted in 72 positive responses. Of these seven were excluded due to recent bereavement and 27 were either contacted but unable to attend on the date of the group meeting or agreed to attend, but did not actually attend. Thirty eight individuals eventually participated in one of seven groups with members of the general population. Characteristics of participants from the general population are shown in Table 1. Unsurprisingly, given the topic area, there was an over‐representation of older people compared with the general population; there was also a high number of individuals who had been bereaved within the last two years. Although data on caring were not collected specifically, during the early part of the focus group, participants were asked to share their thoughts on end of life care generally, and many spoke about experiences of providing care to family and loved ones. Some participants drew on these various experiences in providing their responses and they seemed to form a major element of their knowledge and understanding of the issues for some participants.

**TABLE 1:** Characteristics of participants from the general population (*n* = 38)

Characteristic	Variable	Number (%)
Sex	Female	24 (37%)
	Male	14 (63%)
Age	18–29	4 (11%)
	30–44	1 (3%)
	45–64	11 (29%)
	65+	22 (58%)
Ethnicity	White British	35 (92%)
	Other	3 (8%)
Self‐assessed health (*n* = 36)	Not good	1 (3%)
	Fairly good	13 (36%)
	Good	22 (61%)
Bereaved within last 2 years (*n* = 36)	Yes	10 (28%)
	No	26 (72%)

#### 3.1.2 Policy makers

Four group meetings were held with policy makers, with a total of 29 participants. Three group meetings were held with exisiting local strategy groups focusing on commissioning and provision of health and end of life care; one meeting brought together experts from across the UK in the field more generally including those with expertize in terms of both local and national policy making. Participants in the policy maker groups held a variety of roles including medical; nursing (hospice and community); service management; consultancy; academic; executive and nonexecutive directors within National Health Service providers and commissioners, and in hospices; involvement in local strategy development; and national policy making. Many participants had more than one role. A fifth group meeting was held with another group of stakeholders, seven hospice volunteers, who considered the same tasks, but who are not policy makers. Quantitative results for the policy maker group are presented both including and excluding this volunteer group.

### 3.2 Weights elicited through the budget pie tasks

**FIGURE 5:**
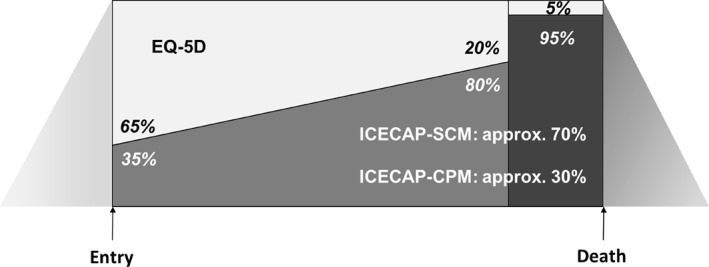
An integrated framework for end of life, populated with relevant weights based on deliberative data collection and a stylized entry to end of life care at 12 months prior to death. The numbers between the light and dark sections represent the relative weights given to health gain versus a good death, while the numbers attached to the ICECAP‐SCM (for those at the end of life) and ICECAP‐CPM (for close persons) represent the approximate relative weights given to the person at the end of life and their families and friends

Weights elicited through the various budget pie tasks are shown in Table 2. Interestingly, the weights for members of the general population and policy makers for the importance that should be given to family and friends are, on average, very similar, at around 30% of the weight. The range of responses is wide, however, for both groups. Only the policy maker group had no participants who were willing to give family and friends a weight of zero. The mean weights that policy makers gave to health gain versus a good death at varying points along the dying trajectory showed a clear gradient away from health gain and toward a good death as an individual gets closer to death. Again, however, there were wide ranges in these responses. Figure 5 shows the populated integrated framework.

**TABLE 2:** Relative weights obtained for tasks A and B postdeliberation, means and ranges

Task A—members of the public and policy makers		
Group	Mean weight (range) given to individual at end of life	Mean weight (range) given to close persons
Members of the general population	70.2 (25–100)	29.8 (0–75)
Policy makers (exc volunteers)	67.3 (25–90)	32.7 (10–75)
Policy makers (inc volunteers)	69.4 (25–90)	30.6 (10–75)

Task B—policy makers excluding volunteers		
Time before death	Mean weight (range) given to health gain	Mean weight (range) given to a good death
12 months	63.3 (0–100)	36.7 (0–100)
6 months	47.9 (0–80)	52.1 (20–100)
1 month	22.1 (0–60)	77.9 (40–100)
A few days	6.3 (0–40)	93.7 (60–100)

Task B—policy makers including volunteers		
Time before death	Mean weight (range) given to health gain	Mean weight (range) given to a good death
12 months	62.7 (0–100)	37.3 (0–100)
6 months	46.9 (0–80)	53.1 (20–100)
1 month	20 (0–60)	80 (40–100)
A few days	5.3 (0–40)	94.7 (60–100)

### 3.3 Insight from deliberation

#### 3.3.1 Persons at the end of life versus their close persons: from one extreme to the other

##### 3.3.1.1 Focus on close persons

In all focus groups, arguments were made in favor of supporting family and friends. The reasons for this differed subtly across the different types of focus groups. An argument made strongly in both samples was around the importance of the role of family and friends in providing care. The longer term impacts on close persons as well as issues around bereavement and communication were both mentioned in both types of group, but seemed to be more important in the policy maker groups. An argument that those at the end of life could no longer really benefit, was made only in the general public groups.

The reason most consistently seen as important for giving weight to benefits for family and friends, discussed across all groups, was the role of family and friends in caring for those at the end of life. In the policy maker groups there was a greater focus on the role of close persons in potentially avoiding crises as the patient moved close to the end of life, while among the general public groups there was a particular focus on the importance of respite care.

PD6: … you need to be able to support the people around that individual in order to avoid emergency admissions and stress calls to GPs… [20 tokens to family and friends; 80 tokens to the person at the end of life]

GC3:… it's having the money for the carers to come into the house to give you support … do some shopping or just catch up, you need that break then you need a carer to sit in with your loved one… [30 tokens to family and friends; 70 tokens to the person at the end of life]

For many participants the impact on family and friends of the death of their close person provided sufficient reason to give a positive weighting to benefits for this group. These impacts were discussed in much greater depth among the policy maker groups, where they were related to the general wellbeing of family and friends, the potential for worsened health and negative economic impacts.

PD8:… thinking about complicated bereaved, people with children, we know that if this has got complex grief, that has implications for mental health for the child etc. in later life… so thinking of the burden on the economy in the future [initial values: 30 tokens to family and friends; 70 tokens to the person at the end of life]

Other reasons for giving weight to family and friends noted in some of each of the policy maker and general public groups were the importance of communication and providing bereavement support. A more limited view, noted in two of the general public groups, was that bereavement support would or should come from the close person's informal support networks.

PA6: …what swung it more towards 75[tokens to family and friends]:25[tokens to the person at the end of life] was… particularly around communication… a lot of misunderstanding comes out of not communicating effectively…

PD5: … pre and post bereavement service… the burden on that family if that was the major bread winning… the suffering might not just be the suffering emotionally and physically… [30 tokens to family and friends; 70 tokens to the person at the end of life]

GC5:… they can get their emotional support through their own relatives. [25 tokens to family and friends; 75 tokens to the person at the end of life]

Some participants in the general public groups felt that the potential benefit to those at the end of life was limited, given their short life expectancy, and allocated their tokens accordingly.

GD67: … at the end of the day, thinking about the really big picture, do we put our resources to people who are going to die? [40 tokens to family and friends; 60 tokens to the person at the end of life]

##### 3.3.1.2 Focus on close persons to help patient

While some participants had a desire to focus on family and friends for their own sake, there were others who reasoned that focusing on family and friends was primarily concerned with the extent to which that support would benefit the person at the end of life.

GE68:… as you're dying the one thing you're worrying about is your family and friends and whether they're getting the sort of support they need. I mean for a lot of people that'd be the case… [20 tokens to family and friends; 80 tokens to the person at the end of life]

##### 3.3.1.3 Joint focus

Some participants in both general public and policy maker groups talked about the very intertwined nature of care at the end of life, which meant that they took a joint focus to the weighting of patients and close persons. Some participants responded to this issue by splitting the tokens equally between the patient and the close persons, while others refused to provide any weighting at all.

PA3: In my experience what the patient often wants is to make sure their family are okay. And, actually, by enabling that you relieve a lot of anxiety for the person that's actually dying. And again, if we don't get it right in terms of that person that's dying, the impact for the people that are left behind can be huge… [50 tokens to family and friends; 50 tokens to the person at the end of life]

GA1: Because I think… one party gets the support it reflects on the other? And if that party gets support it reflects on other? it's almost shared… I put fifty fifty…

##### 3.3.1.4 Focus on patient to help close persons

In around half of the general public and policy maker groups, the argument was made that the “knock‐on effect” of focusing on the person at the end of life was a reason to give less weight explicitly to impacts for family and friends and more weight to the person at the end of life, as this would mean that the needs of the family and friends would also be addressed, albeit indirectly.

GC6:… I'm thinking well, if I'm confident that you are looking after my loved one I can get on with the rest, because that also takes care of me and takes away a lot of the fear and concern … so I've put ninety five‐five… [5 tokens to family and friends; 95 tokens to the person at the end of life]

PB1: …if the impacts around what happens with the patient and how they're supported is done right, it will have a knock on effect

##### 3.3.1.5 Focus on patients

Many participants across both types of group expressed the view that the patient should be the most important focus in decision making. This seemed largely to relate to the intrinsic importance of the person who was experiencing the end of their life, with terms such as “first,” “forefront,” “paramount,” “ultimate” and “most important” being used by participants. For some of those in the policy maker groups, however, this focus was also clearly related to their perceptions of their own role.

GD62: I put 99 to the people at the end of their life and one to family… I think first and foremost the priority should always be the person at the end of their life, and providing the best circumstances, care for them…

PB1: I put 90/10…. ultimately, you know, our role is, is to make sure the person gets the best care.

Arguments were also made against giving weight to family and friends. In some cases this related to a view that family and friends would ultimately cope with their experience, and in others to a view that it was inappropriate to divert resources from the person at the end of life for family support that was “a nice touch” but not essential; here, participants talked about services such as providing “nice” places for family to stay or rest, and offering them refreshments.

GE64:… if the treatment being given to the dying person was all that it should be, then the friends and family … would be fine [ticked ‘no’ to giving support to family and friends]

PD3:… if it's about bereavement support and having a nice room… [that] is quite a lot of money that's not going towards… the care provision and services for the patient that's dying… that would then impact on them. [10 tokens to family and friends; 90 tokens to the person at the end of life]

#### 3.3.2 ”Health gain” versus “a good death”: weighting on a sliding scale

Most participants across all focus groups based their answers on some sort of shift between “health gain” and “a good death” over time, akin to a sliding scale. Although this was an almost universal approach, the points on these sliding scales differed across participants, and they were not necessarily linear. Only two participants, from one group, had a very different set of values, weighting entirely toward a good death at all time points on grounds of inevitability and costs of care that would result in minimal health gain (particularly in terms of life expectancy).

PA6:… if at 12 months you know that you're going to die and there's nothing anybody can do about it then I think as soon as you reach that point you should be focusing on a good death rather than health gain… [All time points: 0 health gain; 100 end of life]

The primary reason for a sliding scale was to take account of the importance of different aspects at different times as the individual moved forward on the trajectory toward death, although these needs were interpreted differently by participants.

PA8: 50:50 at the start because, twelve months, you still want to be able to get around and talk about more the emotional aspects… but obviously you still need to focus on where you want to be in the next twelve months; where you want to die, what would help, what you need to get in place. Then I did it on a sliding scale so eventually I went down to 90:10 towards the end. [Initial values ‐ 12 months: 50 health gain; 50 end of life. 6 months: 40 health gain; 60 end of life. 1 month: 20 health gain; 80 end of life. Few days: 10 health gain; 90 end of life.]

PC3: At 12 months to go 80/20, and then six months to go 75/25, because someone's still very much a living person. And then at one month 50/50 and then at last couple of days 40/60, more because of an individual being a living person even whilst they're in the dying phase…

### 3.4 Reasons for a sliding scale

A number of specific reasons for a sliding scale were noted. The first was the uncertainty that surrounds the expected time of death, and the idea that a twelve‐month prognosis might be optimistic especially in some long‐term conditions.

PD3: Particularly with the long term … we can't define when they're going to die so we need to put along the good death bit alongside the living the best you can bit… [12 months: 70 health gain; 30 end of life. 6 months: 70 health gain; 30 end of life. 1 month: 20 health gain; 80 end of life. Few days: 20 health gain; 80 end of life.]

A second reason was the need to plan for a good death as an individual reached increasing acceptance about their condition. The need for openness was seen as important in this context.

PA2:… I think my sliding scale took that into account as well, you know, that slow drip‐feed of the idea of planning for the end. [12 months: 90 health gain; 10 end of life. 6 months: 60 health gain; 40 end of life. 1 month: 60 health gain; 40 end of life. Few days: 20 health gain; 80 end of life.]

A third reason was the difficulty in drawing a clear distinction between the characteristics of “health gain” and “a good death.”

PA5: … to me a good death is that as long as possible you can, if you want to, look after yourself, be… anxiety‐free, pain‐free is part of a good death but it's also part of health gain as well. So I did find it difficult in that, so I made it sliding scale to sort of cover both of those basis. [12 months: 80 health gain; 20 end of life. 6 months: 60 health gain; 40 end of life. 1 month: 25 health gain; 75 end of life. Few days: 2 health gain; 98 end of life.]

### 3.5 Reasons for choices further away from, and closer to, death

At the early part of the trajectory toward death (i.e., further from death), participants felt that individuals still had the potential for living, still had opportunity to gain health, and that some individuals were not yet ready to accept the inevitability of death. Participants often drew on both clinical and personal experiences in deriving their own weights.

PD1: … thinking about my own mother, right up until the last month of her life, what she wanted to do was live… so it was all about maximising her quality of life rather than the quality of her death. [12 months: 85 health gain; 15 end of life. 6 months: 75 health gain; 25 end of life. 1 month: 50 health gain; 50 end of life. Few days: 20 health gain; 80 end of life.]

PB7: … I've found that at 12 months, they've always got this hope that something will come up… from my experience, it's very hard to get people to open up about what they think will happen in 12 months because they don't want to look that far… [12 months: 100 health gain; 0 end of life. 6 months: 50 health gain; 50 end of life. 1 month: 40 health gain; 60 end of life. Few days: 0 health gain; 100 end of life.]

For most participants the pendulum appeared to swing strongly toward “a good death” once the task moved on to focus on one month before death and a few days before death. At this point, participants talked about the different needs of the person at the end of life, focusing more on issues such as dignity. The view of participants that those at the end of life would have differing needs at this point, meant that they viewed putting money into activities intended to prolong life but that had little chance of success in so doing, as being of little value, and those who had witnessed this type of care, were particularly aware of its potential harms.

PE9: … sometimes some of the staff, the carers and so on, would come out and just say, 'Why did they revive him? Why…’ I sometimes think of the situation as it's a triumph of technology over compassion. Just because we can do it, should we do it? … Every instinct in us says be compassionate. [Unwilling to provide initial scores.]

## 4 DISCUSSION

This paper attempts to integrate methods for measuring capabilities at the end of life into the standard health and care decision making framework that has a health gain focus, using a deliberative process that combined qualitative methods with quantitative tasks. The context explored, focused on the possibilities for integrating the capability for a good death into the health‐gain focused decision making framework used by NICE in the UK. The almost universal support within this study for trading between health gain and a good death, and trading between benefits to the recipient of end of life care and close persons, suggests that economic evaluation in this area should be more aligned with the policy objectives advocated within strategic approaches to end of life care (National Institute for Health and Care Excellence, 2015; National Palliative and End of Life Care Partnership, 2015; NHS England, 2014) and less aligned with standard NICE methods guidance (National Institute for Health and Care Excellence, 2013). Although the work was conducted in the UK context, its broader message is likely to be relevant in other settings where there is a divergence between the methods used in economic appraisal and the stated objectives of care at the end of life.

The work found that both policy makers and members of the general public are willing to weight between capabilities to the person at the end of life and capabilities for those who are close to them (implying a sharing or trading‐off of resources across patients and close persons). Both groups gave higher weights to the person at the end of life, and although there was considerable variation in the weights given by individuals, the mean weight across each group was remarkably similar. Reasoned arguments were made around the choice of weights between the capabilities for each group, including arguments around the joint nature by which these capabilities are generated. The work also found that policy makers are able to weight between health gain and the capability for a good death at various points along the dying trajectory. Although weights again varied on an individual basis, almost all participants weighted on what they often referred to as a “sliding scale” in which greater weight was progressively given to the capability for a good death as the end of life came closer. Again, reasoned arguments were made, which often focused on the differing needs of individuals as they came closer to death.

This is the first study of this type to have been undertaken, so there is little available evidence with which to compare the findings. There is, however, evidence from other clinical areas that the sorts of “knock‐on” effects that participants spoke about here, do occur in health care, such that ameliorating the patient state might also have benefits for those close to them (Al‐Janabi, Van Exel, Brouwer, & Coast, 2016). In terms of method, recent studies within health economics have also found value in using a deliberative approach to enable reflection and collect both qualitative and quantitative data simultaneously (Gansen, Klinger, & Rogowski, 2019; Kinghorn, 2019). There have also been attempts to use qualitative methods to enhance understanding of the values of relevant stakeholders that can be incorporated into decision‐making around health and care services (Campbell et al., 2018; Kinghorn & Coast, 2019). The extent of this work remains limited, however, and methodological issues such as the extent of deliberation that is optimal in these contexts have not been explored.

This exploratory study has both strengths and limitations. Although members of the public were selected through random sampling, in practice those who attended tended to be weighted toward older people, females and white British. Socio‐demographic data were not collected for policy makers, who were recruited purely on the basis of role, but the demographic make‐up of these groups may also have affected the findings. For the first task, the study was able to compare and contrast the views of policy makers and members of the general public. For the second task, only the views of policy makers were obtained. In large part, this was due to another lengthy task that members of the general public were asked to undertake, related to values for the ICECAP‐CPM, but it also reflected the complexity of the second task and the expectation that it would be more meaningful to policy makers, who are more likely to be familiar with NICE decision making. The deliberative nature of the task meant that very complex methods of valuation such as discrete choice experiments, where values are obtained indirectly rather than directly, would have been inappropriate; it was decided that for this exploratory work, the strengths of using a deliberative approach outweighted the limitations of having to use simpler valuation tasks. In practice, the budget pie task worked well and participants were, for the most part, able to comprehend and complete the tasks. The deliberative nature of the valuation process means that the reasoning behind the valuations was also captured. The use of EQ‐5D, which is a relatively narrow measure of health gain was both a strength (enabling a clearer division between the two areas) and limitation (in its lack of breadth), but was dictated by the UK policy setting, where this is the recommended measure for use in decision making.

While this was a small scale deliberative study, it is clear that both policy makers and the general public, on balance, assign positive value to taking account of the impact of care at the end of life on the capabilities of close persons in decision making. Similarly, policy makers, on balance, feel that policy should take account of the capability for a good death in decision making as individuals approach the end of life, and should not focus only on health gain. NICE currently acknowledges that end of life care is a special case, but corrective measures (which have implications for the budgetary threshold) are currently only applied in rare cases of life extension. The work reported in this paper suggests that, in addition to the threshold, the current maximand is inappropriate within the context of supportive end of life care; economic evaluation should take account of the benefits to close persons of supportive end of life care, as well as the importance of nonhealth outcomes, particularly as the person comes toward the very end of life. Adjusting the maximand in this way would imply a greater allocation of resource toward supportive care and close persons. While this work provides some early quantitative weights that could be used to help to design appropriate forms of economic evaluation that incorporate a broader set of objectives, these weights are still exploratory. It would, therefore, also be appropriate to conduct sensitivity analysis around these weights to account for potential variation in the wider population; this is particularly important given that the current COVID‐19 situation has publicized issues around some aspects of end of life, such as the inability of close persons to be with the person at the end of life, which may alter these values.

Further methodological research to inform the basis for assessing the cost‐effectiveness of interventions at the end of life is urgently required. More in depth deliberative work, with longer sessions and more opportunity for in‐depth discussion would be beneficial in allowing arguments to be discussed more fully. Larger scale studies with more representative populations would also be valuable. Further work is also needed to consider the appropriate threshold for maximands that incorporate values that go beyond health. Nevertheless, this work provides a strong starting point for an approach to incorporating broader considerations into economic evaluation of end of life care. Using an integrated capability framework to conduct cost‐effectiveness studies and inform choices about the interventions that will be funded at the end of life would enable these decisions to capture more of what matters to both policy makers and the general public; ultimately, it should enable the funding of interventions that more closely reflect the values within society.

## CONFLICT OF INTEREST

Joanna Coast is one of the developers of the ICECAP suite of capability wellbeing measures.

## Data Availability

Data are unable to be shared as, at the time of data collection, consent was not obtained to share data beyond the research team.
